# Bulk De Novo Mitogenome Assembly from Pooled Total DNA Elucidates the Phylogeny of Weevils (Coleoptera: Curculionoidea)

**DOI:** 10.1093/molbev/msu154

**Published:** 2014-05-06

**Authors:** Conrad P.D.T. Gillett, Alex Crampton-Platt, Martijn J.T.N. Timmermans, Bjarte H. Jordal, Brent C. Emerson, Alfried P. Vogler

**Affiliations:** ^1^Department of Life Sciences, Natural History Museum, London, United Kingdom; ^2^School of Biological Sciences, Centre for Ecology, Evolution and Conservation, University of East Anglia, Norwich, United Kingdom; ^3^Department of Genetics, Evolution and Environment, University College London, London, United Kingdom; ^4^Department of Life Sciences, Silwood Park Campus, Imperial College London, Ascot, Berkshire, United Kingdom; ^5^The Natural History Museum, University Museum of Bergen, Bergen, Norway; ^6^Island Ecology and Evolution Research Group, Instituto de Productos Naturales y Agrobiología, La Laguna, Tenerife, Canary Islands, Spain

**Keywords:** next-generation sequencing, genomics, MiSeq, mitochondria, phylogenetics, wood-boring

## Abstract

Complete mitochondrial genomes have been shown to be reliable markers for phylogeny reconstruction among diverse animal groups. However, the relative difficulty and high cost associated with obtaining de novo full mitogenomes have frequently led to conspicuously low taxon sampling in ensuing studies. Here, we report the successful use of an economical and accessible method for assembling complete or near-complete mitogenomes through shot-gun next-generation sequencing of a single library made from pooled total DNA extracts of numerous target species. To avoid the use of separate indexed libraries for each specimen, and an associated increase in cost, we incorporate standard polymerase chain reaction-based “bait” sequences to identify the assembled mitogenomes. The method was applied to study the higher level phylogenetic relationships in the weevils (Coleoptera: Curculionoidea), producing 92 newly assembled mitogenomes obtained in a single Illumina MiSeq run. The analysis supported a separate origin of wood-boring behavior by the subfamilies Scolytinae, Platypodinae, and Cossoninae. This finding contradicts morphological hypotheses proposing a close relationship between the first two of these but is congruent with previous molecular studies, reinforcing the utility of mitogenomes in phylogeny reconstruction. Our methodology provides a technically simple procedure for generating densely sampled trees from whole mitogenomes and is widely applicable to groups of animals for which bait sequences are the only required prior genome knowledge.

## Introduction

With the advent of high-throughput next-generation sequencing (NGS) technologies and their ability to generate large amounts of data suitable for genomic assembly, systematists are increasingly adopting such methods to reconstruct complete mitochondrial genomes (mitogenomes) to infer phylogenies across a diverse range of taxa. Such research has provided compelling insights in studies ranging from the investigation of deep-level metazoan relationships (Osigus et al. 2013) to those within single phyla (e.g., Cnidaria; Kayal et al. 2013), orders (e.g., Primates; Finstermeier et al. 2013), families (e.g., Braconidae wasps; Wei et al. 2010), and genera (e.g., *Architeuthis* giant squid; Winkelmann et al. 2013). Mitogenomes have an intrinsic suitability for phylogenetic analysis due to their unambiguous orthology (Botero-Castro et al. 2013), phylogenetic signal at diverse taxonomic ranks (Bernt et al. 2013), broadly uniform rate of molecular evolution (Papadopoulou et al. 2010), and uniparental inheritance consistent with bifurcating phylogenetic trees (Curole and Kocher 1999), even if phylogenetic analyses may be confounded by inconsistencies of the coalescent history near the species level (Funk and Omland 2003) and by lineage-specific compositional and rate heterogeneity at higher hierarchical levels (Sheffield et al. 2009; Bernt et al. 2013; Cameron 2014). In addition, the fact that mitochondrial DNA (mtDNA) is present in multiple copies per cell, facilitating its amplification and sequencing, has undoubtedly contributed to the wide use of mitochondrial markers in phylogeny reconstruction. However, in spite of these advantages, complete mitogenome sequencing has been comparatively labor intensive and costly, resulting in often conspicuously few newly generated mitogenomes per study (e.g., 17 bird mitogenomes in Pacheco et al. [2011], four complete Cnidarian mitogenomes in Kayal et al. [2013], and 1 cockroach and 13 termite mitogenomes in Cameron et al. [2012]). Techniques have almost always included either shot-gun sequencing of expensive multiple-indexed libraries (Botero-Castro et al. 2013) or a target-enrichment step, such as primer walking using standard polymerase chain reaction (PCR) amplification of overlapping fragments (Botero-Castro et al. 2013), long-range PCR followed by either sequencing-primer walking (Roos et al. 2007) or shot-gun sequencing (Timmermans et al. 2010), and hybrid-capture using sheared long-range PCR products as “baits” immobilized on magnetic beads (Winkelmann et al. 2013). Although these techniques can generate full mitochondrial genomes, each of them has limitations that generally restrain the number of taxa or samples that can be incorporated economically within a study.

This study aims to address this sampling bottleneck by testing the possibility of parallel de novo mitogenome assembly from a single library of pooled genomic DNA from a bulk sample consisting of many species. This method has recently been applied to sequencing of environmental samples of arthropods from a rainforest canopy (Crampton-Platt AL, Timmermans MJTN, Gimmel ML, Kutty SN, Cockerill TD, Khen CV, Vogler AP, unpublished data). Here, we apply this technique to investigate the higher level phylogeny of an extremely diverse superfamily of insects, the weevils (Coleoptera: Curculionoidea). Mitogenome sequences in the Coleoptera have to date been accumulated gradually for major lineages, including the four suborders, mostly using Sanger sequencing (Sheffield et al. 2008, 2009; Pons et al. 2010; Song et al. 2010; Timmermans et al. 2010). These studies consistently encountered difficulties in resolving basal relationships in Coleoptera due to apparent compositional heterogeneity (Sheffield et al. 2009; Song et al. 2010) and markedly different rates of molecular evolution (Pons et al. 2010). However, it is not known whether heterogeneity, that confounds deep-level divergences, also affects subclades, for example, at the level of superfamilies and families (Cameron 2014). In addition, the effect of different data partitioning schemes remains to be investigated across taxonomic levels (Cameron 2014).

The Curculionoidea are composed of no fewer than 62,000 described species distributed wherever terrestrial plants grow (Oberprieler et al. 2007). The current higher level classification proposed by Bouchard et al. (2011) recognizes nine extant families, among which the Curculionidae *s. str.* is by far the largest, containing at least 51,000 species in 17 subfamilies and 292 tribes and subtribes. The phylogenetic classification of the weevils was recognized by the eminent beetle taxonomist Crowson (1955) as “… probably the largest and most important problem in the higher classification of Coleoptera ….” Since that time, there have been considerable advances in our understanding of the phylogeny of this group, with significant morphological analyses by Kuschel (1995) and Marvaldi (1997). More recently, molecular data have contributed toward reconstructing weevil higher level relationships, including studies by McKenna et al. (2009), Hundsdoerfer et al. (2009), and Jordal et al. (2011), which each incorporated between two and six gene markers. A recent analysis of 27 weevil mitogenomes using 12 protein-coding genes (Haran et al. 2013) supported the paraphyly of Curculionoidea *s. str*. as currently defined, because the subfamily Platypodinae was recovered in a distant position in a clade with the families Dryophthoridae and Brachyceridae that together were sister to all other Curculionoidea. Although undertaken with limited taxon sampling within the Curculionoidea *s. str.* (18 tribes), this last study also supported the division of the family into two large clades: One comprising the “broad-nosed” weevils (subfamilies Entiminae, Cyclominae, and Hyperinae) and another containing the remaining subfamilies (except for Platypodinae). In the same study, a tRNA^Ala^ to tRNA^Arg^ gene order rearrangement was identified in a cluster of six tRNA genes, located between *nad3* and *nad5*, which appears to be a synapomorphy for the “broad-nosed” weevil subfamilies, further supporting their monophyly. This topology was consistent with that proposed by McKenna et al. (2009), who concluded that the initial diversification of weevils occurred on gymnosperm plants during the Early to early Middle Jurassic.

The Platypodinae is one of several weevil subfamilies that are specialist wood-borers, together with the bark-beetles (Scolytinae) and the subfamily Cossoninae, although other subfamilies also contain xylophagous members (e.g., Molytinae, Cryptorhynchinae, and Conoderinae). The evolution of wood-boring behavior was investigated in detail by Jordal et al. (2011), whose analyses incorporated morphological characters together with molecular data, concluding that both Scolytinae and Platypodinae are derived lineages within the Curculionoidea sensu Oberprieler et al. (2007). However, several important head characters that underpin this relationship are likely to be homoplasious and associated with tunneling habit (Jordal et al. 2011). Thompson (1992) identified distinct characters of the platypodine eighth abdominal sternite and male genitalia, which indicated a distant relationship to Scolytinae and a possible justification for their inclusion in a separate curculionoid family. Therefore, the question about the polyphyly of wood-boring lineages remains open, and the failure of previous mitogenome studies to recover the platypodine and scolytine lineages as monophyletic (Haran et al. 2013) may be due to limited taxon sampling. The issue therefore may only be resolved if Jordal et al.’s (2011) comprehensive taxon sampling of wood-boring lineages could be matched using mitochondrial genomes.

## Results

### Mitogenomic Assembly

**Fig. 1.**
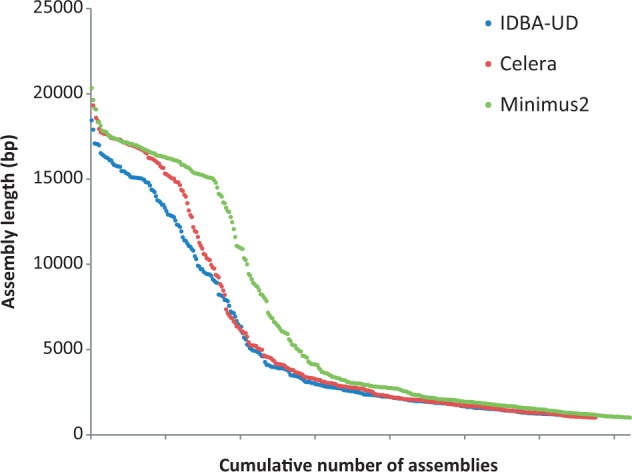
Cumulative distribution of assembly lengths from the Celera, IDBA-UD, and the combined Minimus2-generated assemblies.

**Fig. 2.**
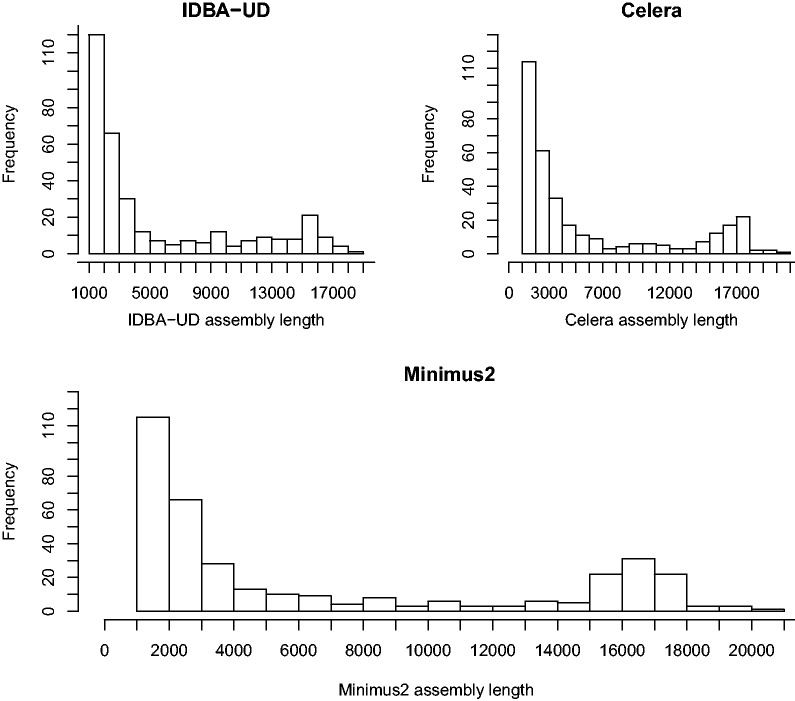
Frequency distribution of assembly lengths from the Celera, IDBA-UD, and the combined Minimus2-generated assemblies.

Specimens were selected to represent a wide taxonomic coverage, and included 173 species from six different families of Curculionoidea, and 16 subfamilies and 104 tribes of Curculionidae. They were acquired from various sources and in different stages of preservation, leading to variable DNA quality, as is common in phylogenetic studies that involve lineages for which DNA-ready material is difficult to obtain. Individual DNA extracts were not characterized in great detail, but based on bait PCR success, they are likely to differ in the degree of degradation and purity. All DNA extracts were included in a single sequencing pool at equimolar concentrations, although for several, including aliquots from 31 specimens already extracted for a previous study (Jordal et al. 2011), the available amount of DNA fell short. Following sequencing with an Illumina MiSeq, approximately 5% of the reads resembled mitochondrial sequences after BLAST filtering (from a total of 18,341,901 paired-end reads obtained in a single MiSeq run). Assemblies constructed with the Celera and IDBA-UD assemblers resulted in 338 and 336 assemblies of more than 1,000 bp, respectively, rising to 361 assemblies when combined using Minimus2. Of these, 105 were more than 10 kb in length and potentially represented (largely) complete mitogenomes. The cumulative distribution of the assemblies by sequence length is shown in figure 1, whereas figure 2 represents the frequency distribution of assembly lengths for each of the Celera, IDBA-UD, and Minimus2 assemblies. The latter produced a shift toward longer contigs, especially for the critical contig length of more than 15 kb that corresponds to the full length of insect mitogenomes. All subsequent analyses were conducted on the Minimus2 assemblies. We were able to newly assemble and identify a total of 92 complete or near-complete mitogenomes comprising at least eight genes, including 75 (43% of all pooled samples) containing the full complement of 15 genes, a further 15 (8.7% of pooled samples) containing more than or equal to 12 genes (supplementary table S1, Supplementary Material online), and two assemblies containing eight and nine genes, respectively. Those falling short of a full-gene complement were mainly lacking the ribosomal RNA (rRNA) genes, in particular *rrnS*, which was the least common gene, present in only 56 of the assemblies, whereas *nad6* and *cytB* were present in all 92 assemblies. A majority of 86 assemblies contained a portion of the noncoding control region, whose exact length is difficult to ascertain because of reduced sequence complexity due to the presence of repeated regions. The mean estimated length of the control region was 1,190 bp, whereas in those 33 mitogenomes that could be circularized, the length varied between approximately 200–2,780 bp (supplementary table S1, Supplementary Material online).

### Identification of Mitogenomic Assemblies Using “Bait” Sequences

**Fig. 3.**
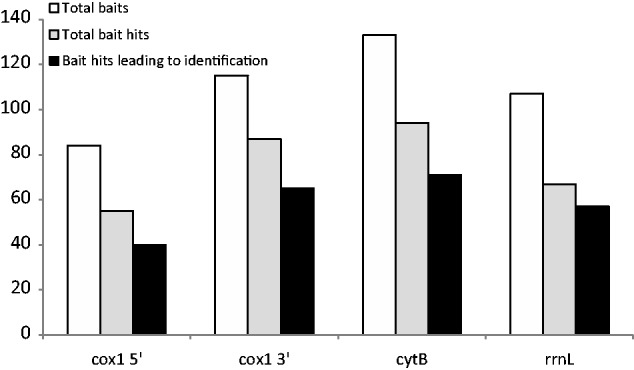
Relative proportions, by gene, of total “bait” sequences available, “bait” sequences with matching “hits” to the assembled genes and matching hits that contributed to a successful mitogenome identification following a BLAST search.

From the set of 361 partial and complete contigs obtained with Minimus2, a total of 163 *cox1* (529–1,560 bp), 154 *cytB* (218–1,147 bp), and 162 *rrnL* (211–1,340 bp) gene sequences were extracted. Sequences from each gene were grouped into libraries and used as queries in a BLAST search against each corresponding bait sequence reference library. The latter was composed of all successful PCR-based sequences from the 173 original DNA extractions and included 84 *cox1*-5′, 115 *cox1-*3′, 132 *cytB*, and 107 *rrnL* sequences (fig. 3). All samples used in the bulk sequencing were represented by at least one bait (36 samples), whereas 42, 57, and 36 samples were represented by two, three, and four bait sequences, respectively. Matching these bait sequences to the 92 long mitogenomic assemblies, 16 assemblies showed a match to one bait, 30 assemblies matched two baits, 32 assemblies matched three baits, and 14 assemblies matched all four baits. Four of the complete and near-complete mitogenomes contained sequences from two nonoverlapping assemblies that each matched at least one bait from the same specimen. Out of the remaining 81 weevil samples, there were 37 instances where baits hit a short contig that was not included in the collection of near-complete or complete mitogenome assemblies, but in 44 instances, the baits did not hit any of the assembled contigs. Additionally, one divergent assembly was rejected because it was found to match Coleoptera other than weevils in the reference database, possibly present in the sample due to a contamination. Supplementary table S2, Supplementary Material online, summarizes the bait-matching identification results, by bait, for each pooled sample, with matching contigs given by their unique number and with reasons for identification failures listed. Overall, the different baits contributed fairly equally to the final identifications, with 56% of all *cox1*-3′ baits leading to a successful identification, 53% of *cytB*, 50% of *rrnL*, and 45% of *cox1-*5′. Proportions of total number of baits, bait hits, and hits leading to assembly identifications by gene are illustrated in figure 3. A further 50 short contigs (1,025–6,437 bp, mean 2,472 bp) matched single baits but were not incorporated in the analyses because they contained only a maximum of four complete protein-coding or rRNA genes each. Their inclusion would have considerably increased the amount of missing data in the matrix.

**Fig. 4.**
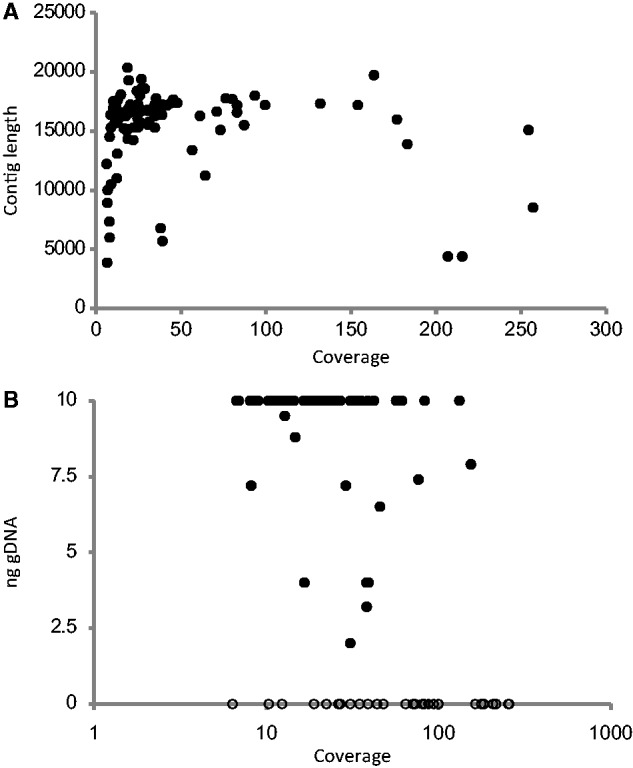
Mean sequencing coverage versus (*A*) assembly (contig) length (bp) and (*B*) approximate mass of genomic DNA in the sample pool, for identified mitogenomic assemblies. Thirty-one samples that were not assayed for DNA concentration are shown at bottom of graph B.

The total number of reads making up each of the 92 mitogenomes (which were made up of 96 separate contigs) was used to calculate the sequencing depth (fig. 4). The majority of sequences showed a 10–50× coverage that generally resulted in contigs of 15–20 kb. Coverage reached over 200× in a few cases, but this did not appear to closely correlate with contig length. For example, two contigs of high coverage were less than 5 kb in length and corresponded to two noncontiguous fragments from the same species (*Dryocoetes autographus*) linked by multiple baits obtained from a single specimen. In addition, read coverage was not closely correlated with the initial DNA concentration in the sequencing pool. Most samples were present at 10 ng, yet their coverage varied by more than an order of magnitude, whereas coverage for samples present at a concentration up to 4× lower varied over the same range (fig. 4). Twenty-one of the 31 nonassayed genomic samples resulted in assemblies of more than or equal to eight genes (of which 17 assemblies contained all 15 genes). We found no taxonomic correlate with sequencing or assembly failure because representatives of all six pooled families and 13 of the 16 included subfamilies of Curculionidae resulted in long assemblies (the three missing subfamilies were represented only by a total of five specimens). Specimen size is also unlikely to be the dominant limiting factor in determining sequencing success because many of the small-sized (∼2–5 mm) Scolytinae produced full assemblies.

### Phylogenetic Analyses

The 92 new assemblies were combined with existing data, for an aligned data matrix of 122 samples and 13,792 positions. Of the final set of mitogenomes, 2 belonged to the family Anthribidae, 5 to Attelabidae, 3 to Brachyceridae, 4 to Brentidae, 4 to Dryophthoridae, 1 to Nemonychidae, and 101 belonged to 67 identified tribes within the Curculionidae, including 19 tribes of the wood-boring Scolytinae. The optimal partitioning scheme was established using PartitionFinder, starting with a total of 39 partitions (41 partitions with the two rRNA genes included) that split all 13 genes (15 in data sets A, C, and E) and three codon positions in each protein-coding gene. PartitionFinder selected five partitions for the “only protein-coding genes” data set and six partitions for the “all genes” data set, whereby the two rRNA genes were grouped with the first codon positions of *nad2*, *nad*3, and *nad6* and the second codon position of *atp8* (table 1). For both data sets, the first and third codon positions on forward and reverse strands were split into separate partitions, whereas all second positions were collapsed into a single partition. Forward and reverse genes mainly differed in base frequencies, with a shift from A to T and G to C in the reverse strand partitions, and rates shifted accordingly (normalized to the time-reversible G-T changes; supplementary fig. S3, Supplementary Material online). The data set containing “only protein-coding genes R-Y coded” resulted in only two partitions, separating first and second codon position for both strands combined (third positions are removed from this data set). The findings are in accordance with previous observations on Curculionoidea that also showed a great improvement in likelihood values when partitioning by both codon position and strand (Haran et al. 2013), reflecting the great differences in codon usage in genes coded on either strand (also see Pons et al. 2010). However, this does not extend to produce differences in variation in amino acid changes, as forward and reverse strands were consistently grouped into a single partition for the data set using second position only and for the R-Y-coded matrix (eliminating first codon synonymous changes).

**Table 1.** Partitioning Schemes and Nucleotide Substitution Models Selected by PartitionFinder for Two Data Sets, According to Gene, and to Codon Position (Numbered 1–3) in Protein-Coding Genes.

Partition	*Nad2*			*cox1*			*cox2*			*atp8*			*atp6*			*cox3*			*nad3*			*nad5*			*nad4*			*nad4L*			*nad6*			*cytB*			*nad1*			*rrnL*	*rrnS*
	1	2	3	1	2	3	1	2	3	1	2	3	1	2	3	1	2	3	1	2	3	1	2	3	1	2	3	1	2	3	1	2	3	1	2	3	1	2	3		
All genes																																									
P1	X										X								X												X									X	X
P2		X			X			X						X			X			X			X			X			X			X			X			X			
P3			X			X			X			X			X			X			X												X			X					
P4				X			X			X						X																		X							
P5																						X			X			X									X				
P6																								X			X			X									X		
Only protein-coding genes																																									
P1	X			X			X			X	X		X			X			X												X			X							
P2		X			X			X						X			X			X			X			X			X			X			X			X			
P3			X			X			X			X			X			X			X												X			X					
P4																						X			X			X									X				
P5																								X			X			X									X		

Note.—Reverse strand transcribed genes are indicated in light gray and the rRNA genes in dark gray. Separate partitions are numbered P1–P6 and allocated positions to each partition labeled X.

The maximum-likelihood (ML) trees were greatly improved using six partitions over an unpartitioned analysis, but the benefit of using a model with 41 or 39 separate partitions was low, as seen from the small additional improvement in the Akaike information criterion (AIC) values (table 2). Interestingly, the improvement in ML from using the partitioned models was very similar whether the trees were obtained directly under the partitioned model or obtained under the unpartitioned model but with the likelihood calculated under partitioning (table 2). Hence, despite the greatly improved likelihood scores after partitioning, the resulting trees differ only slightly in parameters of greatest impact on the likelihood. Indeed, the topologies are little changed between searches using the unpartitioned model, 6-partition model (5-partition model without rRNA genes), and the 41 (39) partition model, and hence, there was only a small increase in likelihood if the simpler model is imposed on the tree obtained with the more complex model.

**Table 2.** ML of Trees under Different Partitioning Schemes.

Data Set	Partitioning Scheme	Topological Constraint	Number of Partitions	Substitution Model	Number of Parameters	Ln *L*	AIC	ΔAIC
All genes	Unpartitioned (one partition)	None	1	GTR	8	−787,773	1,575,562	62,885
	PartitionFinder (six partitions)	On one partition tree	6	GTR	48	−758,061	1,516,219	3,349
	Gene/codon-position (41 partitions)	On one partition tree	41	GTR	328	−756,379	1,513,414	737
	Gene/codon-position (41 partitions)	On six partition tree	41	GTR	328	−756,272	1,513,199	522
	PartitionFinder (six partitions)	On 41 partition tree	6	GTR	48	−758,010	1,516,116	3,439
	Gene/codon-position (41 partitions)	None	41	GTR	328	−756,010	1,512,677	n/a
	PartitionFinder (six partitions)	On one partition tree	6	GTR	48	−758,061	1,516,219	3,542
Protein-coding genes	Unpartitioned (one partition)	None	1	GTR	8	−684,161	1,368,339	34,473
	Gene/codon-position (39 partitions)	On 1 partition tree	39	GTR	312	−666,834	1,334,219	425
	PartitionFinder (5 partitions)	None	5	GTR	40	−668,480	1,337,039	3,173
	Gene/codon-position (39 partitions)	On five partition tree	39	GTR	312	−666,678	1,333,981	115
	PartitionFinder (five partitions)	On 39 partition tree	5	GTR	40	−668,523	1,337,127	3,261
	Gene/codon-position (39 partitions)	None	39	GTR	312	−666,621	1,333,866	n/a
	PartitionFinder (five partitions)	On one partition tree	5	GTR	40	−668,567	1,337,213	3,347

Note.—Trees were obtained under no partitioning, under the six- or five-partition schemes selected by PartitionFinder, and by the maximum number of partitions tested (partitioning by gene and codon position). Each of the resulting trees was then assessed for their likelihood under the alternative models. Note the comparatively small difference in likelihood (ΔAIC) under each partitioning scheme regardless of the model used in the tree search.

**Fig. 5.**
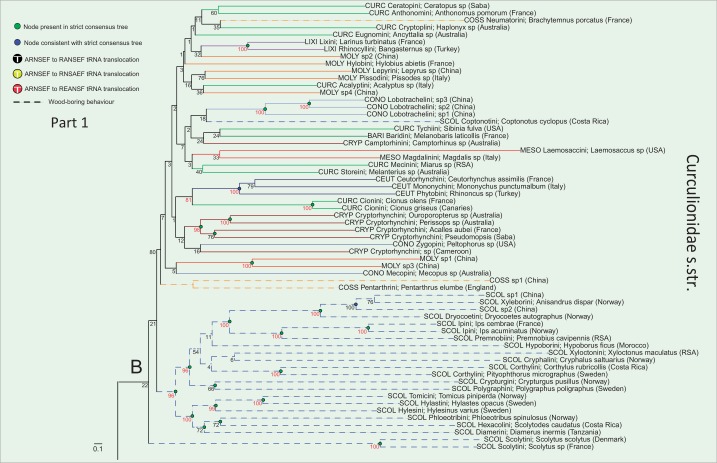

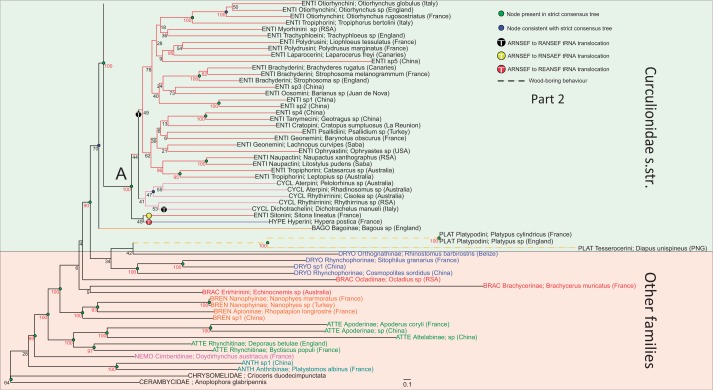
(Parts 1 and 2) ML tree resulting from the analysis of the “all genes” data set partitioned according to the six PartitionFinder partitions (see table 1). Within Curculionidae *s. str*. (sensu Bouchard et al. 2011) branches are colored according to subfamily. Other curculionoid families have their name labels colored by family. Numbers adjacent to nodes are RAxML rapid bootstrap scores, with values more than 80% highlighted in red. The three principal wood-boring subfamilies are represented by dashed branches and the nodes labeled *A* and *B* indicate the two large divisions within Curculionidae referred to in the text. Nodes indicated in green correspond to nodes present in the strict consensus tree and nodes indicated in blue are consistent with it. The positions of the three tRNA rearrangements are indicated. Scale bar represents substitution rate. Family and subfamily codes precede taxa names as follows: Anthribidae (ANTH), Attelabidae (ATTE), Brachyceridae (BRAC), Brentidae (BREN), Dryophthoridae (DRYO), Nemonychidae (NEMO), Bagoinae (BAGO), Baridinae (BARI), Ceutorhynchinae (CEUT), Conoderinae (CONO), Cossoninae (COSS), Cryptorhynchinae (CRYP), Curculioninae (CURC), Lixinae (LIXI), Mesoptillinae (MESO), Molytinae (MOLY), Platypodinae (PLAT), and Scolytinae (SCOL).

ML trees obtained with the various coding schemes (including or excluding rRNA genes, R-Y coding, presence of third codon position; supplementary table S4, Supplementary Material online) also resulted in highly congruent topologies based on strongly supported (>80% bootstrap analysis [BS]) nodes. Figure 5 depicts the best RAxML tree obtained with the “all genes” data set under six partitions. Indicated on this tree are nodes that are retained in the strict consensus of trees obtained from all different treatments of the data, and those nodes unresolved in the strict consensus, that is, the nodes whose resolution is consistent with the strict consensus. Nodes with high nodal support (80–100% BS) occurred throughout the entire span of nodal ages, and this pattern is found across all analyses (supplementary fig. S5, Supplementary Material online). Results obtained from the three additional smaller subsets of data indicate that the trees obtained using the plus- and minus-strand-encoded subsets of genes (supplementary figs. S8 and S9, Supplementary Material online) agree well with the full matrix-derived trees, but importantly, those constructed using only the “bait” sequences (supplementary fig. S6, Supplementary Material online) contain much lower nodal support than any of the mitogenomic trees. This is expected from a data matrix that has much missing data, which consequently does not allow for robust inference of relationships.

The data set also allowed us to address the question about the hierarchical level at which the confounding effects of compositional heterogeneity may be encountered (Sheffield et al. 2009; Song et al. 2010). The χ^2^ test of base heterogeneity (Swofford 2002) revealed that with only one exception (*atp8*) the data are heterogeneous by this test (supplementary table S7, Supplementary Material online). In contrast, the R-Y recoded data, stripped for third positions, indicated that most genes are homogeneous by this test, although not for the concatenated complete matrix. However, the more defensible test of Foster (2004) showed that only *cox3*, *cytb*, and *nad1* are homogenous in composition. Hence, the issues of heterogeneity persist at a much lower hierarchical level than the subordinal and superfamily-level relationships investigated previously (Sheffield et al. 2009; Song et al. 2010).

### Family-Level Relationships

All 15 analyses recovered the monophyletic “ambrosia beetles,” Platypodinae (100% BS) outside the other “true weevils” (=Curculionidae sensu Bouchard et al. 2011), which would otherwise be monophyletic. In most analyses, except those including R-Y-coded protein-coding genes, Platypodinae was placed in the sister clade to the rest of Curculionidae, together with the Dryophthoridae (palm weevils) and the brachycerid genus *Ocladius*, with moderate to strong support for this adelphic relationship (62–95% BS). In all analyses, the monophyletic Brentidae (100% BS) were recovered as the sister taxon to a Curculionidae + Dryophthoridae +Brachyceridae clade with very strong nodal support (100% BS). The sister relationship between the monophyletic (100% BS) Attelabidae (leaf-rolling weevils) and this latter clade plus Brentidae was similarly very strongly supported (100% BS) across all analyses. The Nemonychidae was consistently recovered as sister to the clade containing Attelabidae and all other weevil families mentioned so far. Support for this relationship was very high, ranging from 98% to 100% BS across analyses. The two taxa belonging to the Anthribidae were always recovered as monophyletic (100% BS). Within the Attelabidae, the subfamilies Apoderinae and Rhynchitinae were recovered as monophyletic with BS support of 100% and 83–97%, respectively, across analyses.

### Relationships within Curculionidae *s. str.*

In most analyses, the subfamily Bagoinae, represented only by a single *Bagous*, was recovered as the sister to all other Curculionidae (excepting Platypodinae as noted above), with BS support between 66% and 91%. Similarly, most analyses resulted in the recovery of both a monophyletic Entiminae + Cyclominae + Hyperinae clade (marked A in fig. 5; 100% BS) and a strongly supported sister relationship between this clade and a second clade (marked B in fig. 5) containing all other Curculionidae subfamilies (100% BS). Within the entimine clade, the Entiminae itself is not recovered as monophyletic because the tribe Sitonini is consistently recovered (100% BS) either as sister to the clade containing Hyperinae + Cyclominae + the rest of Entiminae or in a sister clade also containing the Hyperinae (with generally weak nodal support for this relationship). Three entimine tribes are consistently recovered as monophyletic, with strong nodal support; the Otiorhynchini (100% BS), Brachyderini (100% BS), and the Naupactini (100% BS). The tribe Tropiphorini is apparently paraphyletic because a well-supported clade (95% BS), containing two monophyletic Australian members (*Catasarcus* and *Leptopius*), is itself sister to the Naupactini with strong support (96% BS) and is only distantly related to the other Tropiphorini species in the data set (*Tropiphorus*), which is sister to the Otiorhynchini with strong nodal support (100% BS). All Entiminae (except *Sitona*) are marked by an ARNSEF to RANSEF rearrangement in the tRNA cluster, discovered in earlier studies (Song et al. 2010; Haran et al. 2013) and corroborated here (fig. 5). One taxon, *Dichotrachelus manueli*, classified in Cyclominae by Alonso-Zarazaga and Lyal (1999), also possesses this same rearrangement, whereas the remaining Cyclominae taxa possess the common gene order, ARNSEF. *Sitona* and *Hypera* were characterized by unique RNSAEF and REANSF gene orders, respectively, first observed by Haran et al. (2013) and hypothesized to constitute an initial step in the evolution of the derived gene order of the Entiminae. Here, *Hypera* + *Sitona* form a clade that is sister to all others in clade A, whereas the Cyclominae (minus *Dichotrachelus*), not represented in Haran et al. (2013), and exhibiting the ancestral gene order, occupy the next node as sister to the remaining Entiminae characterized by the derived gene order. This demonstrates that the gene order changes in *Hypera* and *Sitona* are independent of those in Entiminae.

Within the second main curculionid clade, the scolytine taxon *Coptonotus* (Coptonotini) is never recovered together with the bulk of the scolytines, which except for Scolytini (monophyletic with 100% BS), are consistently recovered in a clade with moderate to high support values of 66–100%. The scolytine tribes Corthylini and Ipini are always recovered as monophyletic (100% BS support) within this. The following higher level taxa from the second main Curculionidae clade are recovered as monophyletic across all analyses (BS supports follow taxon name): Ceutorhynchinae (100%), Lixinae (100%), Conoderinae Lobotrachelini (100%), and Curculioninae Cionini (100%). The Cryptorhynchini appears to be paraphyletic owing to the presence of a sample (Cryptorhynchini sp. from Cameroon) falling outside the well-supported clade (98% BS) comprising all four other genera analyzed.

## Discussion

### Contig Formation from Pooled Total DNA Sequencing

Our results provide a clear demonstration of economic, efficient and reliable sequencing, assembly, and identification of large numbers of mitogenomes from a pool of total DNA of numerous samples, without any enrichment or PCR amplification. We obtained a complete or near-complete set of protein-coding genes for well over 50% of all samples attempted. Other recent papers attempting to generate full mitochondrial genomes from total DNA either generated a separate library for each taxon (Williams et al. 2014) or pooled only a small number of distantly related taxa (Rubinstein et al. 2013). We have been able to employ the resulting sequence data to reconstruct a higher level phylogeny of the superfamily Curculionoidea that is highly congruent with recent molecular phylogenies and provides additional evidence for the convergent evolution of specialized wood-boring behavior and morphology in weevils. The method has been explored previously for the analysis of bulk insect samples from a forest canopy (Crampton-Platt AL, Timmermans MJTN, Gimmel ML, Kutty SN, Cockerill TD, Khen CV, Vogler AP, unpublished data), applied to nearly 500 individuals from more than 200 species. They found that the assembly of mitogenomes from bulk samples is hampered by substantial differences in DNA concentration for species in the pool, due to variation in both body size and number of specimens representing a species. In addition, intraspecific variation was found to cause difficulties with assembly due to polymorphisms, mirroring the well-known problem with genome assembly from heterozygotes (e.g., Langley et al. 2011). The design of this study was expected to avoid these problems by normalizing the DNA concentration in the pool and by selecting a single individual per species. However, we find that there is no close correlation of sequencing depth and assembly success (fig. 4), in accordance with Crampton-Platt AL, Timmermans MJTN, Gimmel ML, Kutty SN, Cockerill TD, Khen CV, and Vogler AP (unpublished data). Our study excludes the presence of intraspecific variation but indicates that there is a sequencing depth at which assemblers no longer operate optimally, possibly due to the larger numbers of individual sequencing errors contributed by overlapping reads.

A concern of pooled assemblies is the formation of chimeras by the miss-assembly of different mitogenomes. The potential for this is expected to increase if closely related samples that may not differ in conserved regions of the mitogenomes are included in the pool. The prevalence of chimeras was tested using 77 taxa for which multiple baits were available. In many cases, these tests involved both the *cytb* or *rrnL* and the two fragments of the *cox1* gene that map to distant positions in the mitogenome. We did not observe a single case of chimera formation. In addition, the tree topology gave no reason to suggest chimeras, because of the monophyly of the smaller families of Curculionoidea, whereas chimera formation would also have produced great differences in the length of terminal branches, which were not observed.

### Phylogenetic Analysis from Densely Sampled Mitogenomes

Together with existing mitogenome sequences, a total of 120 terminals were included in the phylogenetic analysis. As mitogenome data sets increase with the numbers of taxa needed for dense sampling, this may produce problems with tree searches and model choice. Specifically, the most complex models, such as the amino acid-based CAT model used by Timmermans et al. (2010) that was required for resolving the deep-level relationships within the Coleoptera, are not practical when the number of taxa becomes larger. This raises the question of what is the value of using complex models. Haran et al. (2013) have shown that likelihood trees of weevils can be substantially improved under model partitioning according to 1) codon position and 2) forward versus reverse strand, the latter presumably due to the well-established differences in codon usage on either strand. We conducted a formal analysis to test whether this partitioning scheme by strand and codon captures the most important aspects of the nucleotide variation using the PartitionFinder software, starting from 41 potential partitions of each codon position within each gene. This could be reduced to the codon positions for all genes on either strands, similar to Haran et al. (2013), but maintaining a single partition for the second codon position on either strand, while adding a separate partition for the rRNA genes not included in that study. The use of these six partitions over the full set of 41 partitions led only to a small reduction in likelihood, whereas the unpartitioned models were substantially worse (table 2).

A general difficulty for comparing models is that comparisons are only possible for a single topology but searches under different partitions favor different topologies. We therefore used the optimal trees obtained under no partitioning and the 6- and 41-partition schemes to assess likelihoods of the alternative partitioning schemes on those three topologies. The likelihoods on all trees for the three models were almost identical (table 2), indicating that tree topology is not a major deciding factor for the best model. Taken at face value, the 41 partition wins out over the 6 partition scheme in all three analyses, but the likelihood gain is minor. As likelihood values become very large with the use of numerous whole mitogenomes, AIC values may not be an appropriate approach to avoid overparameterization, unless they are normalized for the total likelihood values (Castoe et al. 2005). We therefore believe the 6-partition scheme is fully adequate. In addition, the practicalities of tree searches on increasingly large data sets from full mitogenomes, as generated with the proposed methodology, also strongly argue for parameter reduction.

Trees obtained from analysis of full mitogenomes were the most robust, but those obtained using the subsets of protein-coding genes resulted in good topological approximations to them (supplementary figs. S8 and S9, Supplementary Material online), suggesting that phylogenetic signal is largely uniform across genes, and is strengthened with additional data. This can be seen by the recovery of certain monophyletic groups such as the Cyclominae only possible with the full matrix. However, trees constructed from the “bait” sequences alone were the least robust, due to both the reduced information content (comparable to the reverse strand genes) and to considerable missing data.

### Implications for the Systematics of Weevils

The close relationship linking Platypodinae with Dryophthoridae, as sister to the Curculionidae *s. str.*, has been demonstrated multiple times (Marvaldi 1997; McKenna et al. 2009; Haran et al. 2013) and indicates that the family Curculionidae, as presently classified, is paraphyletic. The simplified classification system proposed by Oberprieler et al. (2007), recognizing a broader Curculionidae also containing the presently defined Brachyceridae and Dryophthoridae as respective subfamilies (sensu Alonso-Zarazaga and Lyal 1999) would be consistent with our family-level results. Our results strongly support the relationships among the curculionoid families at the base of the tree, which are consistent with most previous molecular analyses, with the exception of the placement of Nemonychidae. This family has previously been suggested to be split off at the most basal node (e.g., McKenna et al. 2009), as opposed to Anthribidae in our results, but our sampling lacks two of the “primitive” weevil families (Belidae and Caridae), prohibiting a definitive conclusion. Our results are also consistent with the previously suggested hypothesis that the Brentidae are the sister family to all the “true weevils,” Curculionidae, if we include Brachyceridae and Dryophthoridae in the latter.

A previously described deep split within the true weevils was confirmed by our substantially increased sampling. One strongly supported clade contains the Entiminae + Cyclominae + Hyperinae and represents the monophyletic and diverse “broad-nosed” weevils, so named because of their relatively short and blunt rostrums. Rearrangements within the cluster of six tRNA genes are restricted to this clade, even with our increased taxon coverage, further supporting its distinctiveness. The cyclomine genus *Dichotrachelus*, containing the same RANSEF rearrangement as all other Entiminae (except *Sitona*) in our analysis, has been treated as belonging to the Entiminae by some authors (Meregalli and Osella 2007) on morphological grounds. Combined with the low nodal support for its inclusion in a monophyletic Cyclominae (<50% BS), our tRNA rearrangement data are consistent with this opinion. The second clade containing all other curculionoid subfamilies, with the exception of Bagoinae, which is placed outside of the two main clades, is much less satisfactorily resolved, with only two of its constituent subfamilies (Lixinae and Ceutorhynchinae) being monophyletic. It contains a number of very large subfamilies including the Curculioninae, Molytinae, Baridinae, Cryptorhynchinae, and Conoderinae, whose relationships remain obscure due to a lack of strong nodal support. Although the recovery of two tribes within this group being monophyletic (Lobotrachelini and Cionini) is encouraging, to further investigate the confusing topology of this clade, significantly more representative taxon sampling will be required. Indeed, limitations in taxon sampling are often cited as potentially limiting factors in higher level phylogenetics (Franz and Engel 2010), and this is certainly an important consideration in such a large group as the Curculionoidea.

An interesting finding is that strong nodal support spans the full depth of the tree and differing taxonomic ranks (families, subfamilies, and tribes; supplementary fig. S5, Supplementary Material online). This pattern was seen in analyses of all data sets and under all partitioning models. A potential criticism of mitochondrial sequence data is that due to accelerated evolutionary rates, saturation of sites may obscure or distort phylogenetic signal at deeper nodes (Talavera and Vila 2011). It is clear from our data that at least at the intrasuperfamily level in weevils, this is not necessarily the case, with phylogenetic signal being evenly distributed across the estimated 170 My diversification history of the weevils (McKenna et al. 2009).

### Evolution of Wood-Boring Behavior

The wood-boring weevil subfamilies are highly adapted to excavate galleries, either subcortically or in woody tissue, and feed on ligneous matter directly or cultivate symbiotic fungi in the tunnels as a food source, and for this reason, many are widespread pests of forestry (Oberprieler et al. 2007). The taxon density of the current analysis nearly matched the extensive sampling of the wood-boring groups by Jordal et al. (2011), a study that is the basis for suggesting their close affinity. However, in contrast to Jordal et al. (2011), our results support the conclusions of Haran et al. (2013) and McKenna et al. (2009), indicating that wood-boring lineages are clearly not monophyletic, with Platypodinae consistently retrieved as closely related to the Dryophthoridae (and Brachyceridae) in a clade sister to all other Curculionidae sensu Bouchard et al. (2011). Although our analyses recovered neither the Scolytinae nor the Cossoninae as monophyletic, and they were never recovered as sister taxa or nested within the same clade, we cannot confidently conclude as to the relationship between them because only a series of weakly supported nodes separate the cossonine taxa and *Coptonotus* from the rest of the Scolytinae. The latter genus is interesting for consistently not being recovered in our analyses within the generally well-supported Scolytinae clade (excepting Scolytini). Based on morphological characters, *Coptonotus* has been considered to be a transitional taxon between Platypodinae and other Curculionidae (Jordal et al. 2011) or alternatively as an intermediate form between Cossoninae and Scolytinae (Thompson 1992), while also containing morphological characters linking it with Cossoninae. Thompson (1992) has suggested a close relationship between Coptonotini and the scolytine tribe Hylastini based on structures of the aedeagus. However, our results argue against this because the Hylastini sample (*Hylastes opacus*) was retrieved with strong support as the sister of Tomicini, and this clade itself was strongly supported as sister to the Hylesini, within the main Scolytinae clade.

## Conclusions

We have demonstrated the relative ease of efficiently and economically obtaining a large number of mitogenome DNA sequences from a pooled mixture of DNA extracts, without the need for enrichment or species-specific tagging prior to genome pooling. Mitogenome sequences are confidently identified to specimen with a limited amount of prior mtDNA sequence data for each sample and exhibit no error with regard to these bait sequences. Our mtDNA genome data yield phylogenetic relationships that are highly congruent with prior expectations and provide phylogenetic signal with robustly supported nodes across a broad range of lineage divergence times and taxon diversity, from family level to generic level, which are consistent across different data partitioning schemes.

It is evident that the efficiency of our approach will be a function of the relative concentration of mitochondrial to nuclear DNA within a focal group. The average coleopteran genome size is estimated to be approximately 0.65 Gb ± 0.05 (http://www.genomesize.com, last accessed May 10, 2014). Under the assumption that the copy number of mtDNA genomes does not differ substantially across organisms, our approach should be of broad utility within insect phylogenetics where mean nuclear genome size is estimated to be 1.22 Gb ± 0.05. However, it may be less efficient for taxa with larger average nuclear genome sizes (e.g., crustaceans: mean nuclear genome size = ∼4.45 Gb ± 0.45). A further consideration for the implementation of our approach is taxon sampling and the mitogenomic assembly pipeline. Our sampling for the higher level taxonomic relationships within the Curculionoidea provides little challenge for the pipeline, as mtDNA genomes sampled from different genera exhibit high DNA sequence divergence. Genome divergence facilitates genome reassembly from a mixed pool of genome fragments, and the pipeline efficiency will eventually be compromised as mtDNA genome relatedness increases. Our data suggest that this limit lies somewhere below an uncorrected divergence of 10% for *cox*1 and *cytB* that characterizes the two species of *Cionus* (*C. olens* and *C. griseus*) included in our sampling. To ascertain genome relatedness thresholds for the reassembly pipeline, simulation analyses can be employed. However, it is important to point out that as NGS technology and read lengths improve, relatedness thresholds will also become more favorable.

## Materials and Methods

### Taxon Sampling, DNA Extraction, and Quantification

Throughout this study, the most recent higher level classification of Curculionoidea, proposed by Bouchard et al. (2011) is adhered to, whereas the assignment of genera to higher taxa follows the catalog of Alonso-Zarazaga and Lyal (1999). DNA was extracted from each ethanol-preserved specimen individually using DNeasy blood and tissue extraction kits (Qiagen). The concentration of double-stranded DNA (dsDNA) in most extractions (139 of 173) was assayed on a Qubit fluorometer using a dsDNA high-sensitivity kit (Invitrogen).

### “Bait” Sequence PCR

Standard PCR reactions to amplify four different fragments of mtDNA (*cox1* 5′ “barcode region,” *cox1* 3′ region, *rrnL* and *cytb*) were undertaken for each of the 173 samples. Primers and reaction conditions are listed in supplementary table S10, Supplementary Material online. PCR products were first cleaned with a size-exclusion filter (Merck Millipore) and then Sanger sequenced; the resulting bait sequences were subsequently employed to identify mitogenomic assemblies in the manner detailed below.

### Sample Pooling and Sequencing

To minimize the effects of DNA concentration on assembly success across all samples, approximately equimolar quantities of genomic DNA from each of the samples were pooled, aiming for 10 ng of dsDNA per sample, resulting in a DNA pool of approximately 1.5 µg. This calculation did not consider 31 samples which were not quantified because of limited sample volume. For each of these, a fixed volume of either 5 or 8 µl was added to the pool. Based on the findings of Crampton-Platt AL, Timmermans MJTN, Gimmel ML, Kutty SN, Cockerill TD, Khen CV, and Vogler AP (unpublished data), where longer insert size was found to result in longer mitochondrial contigs, a TruSeq library was prepared from the pool aiming for an insert size of 800 bp. Quantification of the final library indicated that the average insert size was 790 bp, and this was sequenced on a single Illumina MiSeq run (500-cycle, 250 bp paired-end reads, version 2 reagent kit).

### Mitogenomic Assembly Pipeline

**Fig. 6.**
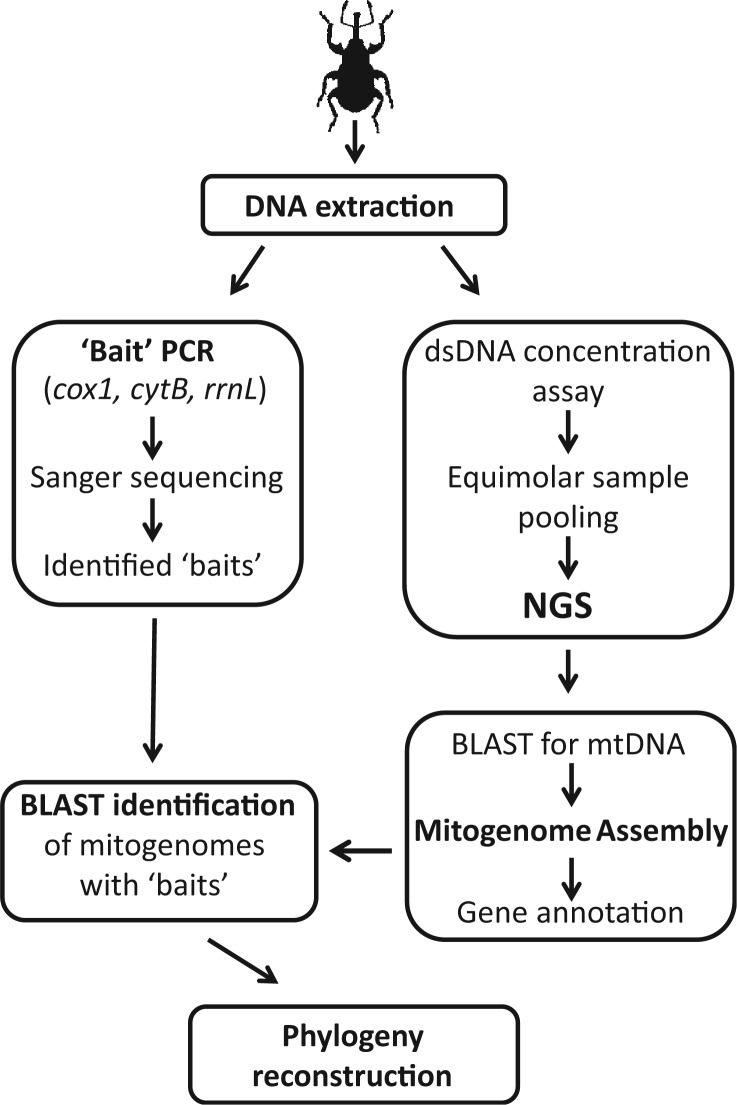
Schematic flowchart of the principal steps for the bulk de novo assembly of mitogenomes and identification with PCR-amplified “bait” sequences.

The bioinformatics assembly pipeline used in this study was developed by Crampton-Platt AL, Timmermans MJTN, Gimmel ML, Kutty SN, Cockerill TD, Khen CV, and Vogler AP (unpublished data) and is followed here with minor modifications. A list of the software required (most freely available) is given in table 3 and a schematic overview of the principal steps is presented in figure 6. In brief, the raw data were trimmed of adapters using Trimmomatic (Bolger et al. 2014), and putative mitochondrial reads were identified in a BLAST search (Altschul et al. 1990) against a custom reference database of 258 Coleoptera mitogenomes (*E* = 1e−5; no restriction in length overlap) (Timmermans MJTN, Barton C, Dodsworth S, Haran J, Ahrens D, Foster PG, Bocak L, and Vogler AP, unpublished data). The extracted mtDNA reads were subjected to whole-genome shot-gun assembly using Celera Assembler (Myers et al. 2000) and IDBA-UD (Peng et al. 2012), and the resulting contigs were filtered again for mtDNA hits against the Coleoptera reference library for sequences of more than 1,000 bp overlap at *E* = 1e−5. Both assemblies were merged using Minimus2 (Sommer et al. 2007) to combine overlapping sequences from both assemblers into longer scaffolds.

**Table 3.** List of Software Used for the De Novo Assembly and Analysis of Mitogenomes, with their Main Function and Source URL.

Software	Function	URL^a^
FastQC	NGS quality assessment	http://www.bioinformatics.babraham.ac.uk/projects/fastqc/
Trimmomatic	Adapter trimming	http://www.usadellab.org/cms/index.php?page=trimmomatic
Celera	Genome assembly	http://sourceforge.net/apps/mediawiki/wgs-assembler/index.php?title=Main_Page
IDBA-UD	Genome assembly	http://i.cs.hku.hk/∼alse/hkubrg/projects/idba_ud/
Minimus2	Merging sequence sets	http://sourceforge.net/apps/mediawiki/amos/index.php?title=Minimus2
Prinseq	Sequence quality control	http://edwards.sdsu.edu/cgi-bin/prinseq/prinseq.cgi
COVE	tRNA annotation	http://selab.janelia.org/software.html
FeatureExtract	Gene extraction	http://www.cbs.dtu.dk/services/FeatureExtract/
Geneious	Gene annotation/sequence editing	http://www.geneious.com/
MAFFT	Sequence alignment	http://mafft.cbrc.jp/alignment/software/
BLAST	Local alignment search	http://blast.ncbi.nlm.nih.gov/Blast.cgi
PartitionFinder	Partitioning scheme selection	http://www.robertlanfear.com/partitionfinder/
CIPRES	Phylogenetic analysis server	http://www.phylo.org/
RAxML	Maximum-likelihood phylogenetic analysis	http://sco.h-its.org/exelixis/software.html
“APE” package in R	Phylogenetic analysis	http://ape-package.ird.fr/

^a^All URLs were last accessed on May 10, 2014.

To investigate the relationship between the number of generated sequencing reads and assembly success, all reads were mapped onto the obtained contigs using Geneious, allowing for 2% mismatches, a maximum gap size of 3 bp and requiring a minimum overlap of 100 bp. Annotations of each assembly were conducted by first mapping tRNA genes with COVE (Eddy and Durbin 1994), after which the intervening protein and rRNA coding genes were extracted with FeatureExtract 1.2 (Wernersson 2005). To identify these genes, the resulting sequences were mapped to the *Tribolium castaneum* mitogenome (GenBank accession number NC_003081) using Geneious and were afterward exported, by gene, into separate FASTA files. Sequences of less than one-third of total gene length were discarded.

### Identification of Mitogenomic Assemblies Using “Bait” Sequences

To identify the mitogenomic assemblies by association with their respective originating specimen, BLAST searches were conducted for each bait sequence reference against all corresponding gene sequences extracted from the mitogenome assemblies (separately for *cox1-*5′ and 3′ regions, *cytB* and *rrnL*). Only hits with 100% pairwise identity and more than 100 bp overlap were considered a successful identification. Where multiple bait sequences from a single specimen were available, each bait was checked to have hit the same long assembly unequivocally to test for possible chimeras. If baits from a single specimen matched multiple, nonoverlapping assemblies, they presumably correspond to the same incompletely assembled mitogenome. These assemblies were combined and retained if they included eight or more genes in total. Once mitogenomic assemblies were identified, the tRNA gene order in the cluster of six tRNA genes located between *nad3* and *nad5* was visually recorded.

### Sequence Alignment and Data Set Concatenation

The sequences for the genes *nad5*, *nad*4, *nad4L*, and *nad1*, which are transcribed on the reverse strand of the mitochondrial genome, were reverse complemented prior to alignment. Twenty-eight additional curculionoid mitogenome sequences were obtained from GenBank (primarily those generated by Haran et al. [2013]; supplementary table S1, Supplementary Material online) to maximize taxon sampling. Two members of Chrysomeloidea were included as outgroups, following Haran et al. (2013). The combined sequences from each of the separated 13 protein-coding and two rRNA genes were individually aligned using the MAFFT 7.0 online server, under the FFT-NS-I slow iterative refinement strategy (Katoh et al. 2002). Alignments were thereafter checked manually in Geneious for quality and to ensure that protein-coding genes were in the correct reading frame. Genes were concatenated together to make six different data matrices as follows: All genes (A), only protein-coding genes (B), all genes with third codon positions removed from protein-coding genes (C), protein-coding genes only with third codon positions removed (D), all genes with third codon positions removed from protein-coding genes and first codon positions R-Y coded (E), and only protein-coding genes with third codon positions removed and first codon positions R-Y coded (F).

### Phylogenetic Analyses

Each of the six data sets was analyzed under the ML optimality criterion using RAxML 7.6.6 (Stamatakis 2006) run on the CIPRES web-based server (Miller et al. 2010). To assess nodal support, a rapid BS with 1,000 iterations was run in parallel with tree-building. The data sets were each analyzed both partitioned by gene and unpartitioned (i.e., a single partition). Additionally, three of the data sets (A, B, and E) were first tested using PartitionFinder (Lanfear et al. 2012) to objectively select the best-fitting partitioning scheme and model of molecular evolution for each alignment. This was performed using the Bayesian Information Criterion from an initial partitioning of each of the three codon positions for each amino acid-coding sequence and each rRNA gene being separate partitions. The resulting ML trees were made ultrametric using the *chronos* function of the APE package in R (Paradis et al. 2004), which uses penalized likelihood to fit a chronogram to a phylogenetic tree (Paradis 2013). To obtain a measure of the suitability of the mitogenomic data to robustly support relationships across different nodal ages (putative taxonomic ranks), we investigated the distribution of nodal support across trees by calculating the branch length from the root for each node using a custom R script and plotting this against its respective RAxML BS support. We also constructed a strict consensus tree from the 15 ML trees to visualize the distribution of consistent nodes across all our analyses. We performed additional RAxML analyses on data sets A and B partitioned by gene and separate codon positions for each protein-coding gene (41 and 39 partitions, respectively) and various RAxML analyses on these two data sets with different combinations of partitioning schemes and topological constraints, as summarized in table 2, in order to calculate the AIC as a means for preferred model selection (Posada and Buckley 2004).

To investigate how successfully subsets of the full-data matrix were able to reconstruct the phylogeny, we also analyzed (using RAxML with data partitioned by gene and by PartitionFinder-derived partitions) three additional data sets, composed of: 1) only the reverse-transcribed protein-coding genes (*nad5*, *nad4*, *nad4L*, and *nad1*), 2) the remaining nine forward-transcribed protein-coding genes, and 3) only the available “bait” sequences. The latter analysis was undertaken to ascertain whether there is any benefit in assembling the mitogenomes for phylogeny reconstruction, over the PCR sequences alone.

Compositional heterogeneity was assessed on the protein-coding genes using the χ^2^ statistics (Swofford 2002). The resulting *P* value is the probability that the data are homogeneous and is considered significant when less than 5%. This test suffers from a high probability of Type II error because the test assumes independence of the data, which they are not. We therefore also used the test of Foster (2004), which uses simulations based on the ML tree, model, and data size to generate a valid null distribution of a χ^2^ value from the original data. The ML tree for the concatenated data was used in all cases when assessing heterogeneity in each gene, with any missing taxa pruned off. Model parameters and branch lengths were reoptimized under a GTR + G model.

## Supplementary Material

Supplementary tables S1, S2, S4, S7, and S10 and figures S3, S5, S6, S8, and S9 are available at *Molecular Biology and Evolution* online (http://www.mbe.oxfordjournals.org/).

Supplementary Data
