# Supplementary material for: Bulk De Novo Mitogenome Assembly from Pooled Total DNA Elucidates the Phylogeny of Weevils (Coleoptera: Curculionoidea)
Source: Mol Biol Evol. 2014 May 6;31(8):2223–37. doi: 10.1093/molbev/msu154 (PMC4104315; doi:10.1093/molbev/msu154)
Supplement: Supplementary Data [file supp_31_8_2223__index.html]

Bulk de novo mitogenome assembly from pooled total DNA elucidates the phylogeny of weevils (Coleoptera: Curculionoidea) — Bulk De Novo Mitogenome Assembly from Pooled Total DNA Elucidates the Phylogeny of Weevils (Coleoptera: Curculionoidea) — Bulk De Novo Mitogenome Assembly from Pooled Total DNA Elucidates the Phylogeny of Weevils (Coleoptera: Curculionoidea) — Bulk De Novo Mitogenome Assembly from Pooled Total DNA Elucidates the Phylogeny of Weevils (Coleoptera: Curculionoidea) — Supplementary Data 

# Bulk De Novo Mitogenome Assembly from Pooled Total DNA Elucidates the Phylogeny of Weevils (Coleoptera: Curculionoidea)

## Supplementary Data

files

**Files in this Data Supplement:**

- Supplementary Data - pdf file
- Supplementary Data - pdf file
- Supplementary Data - xlsx file
