# Supplementary material for: Bulk De Novo Mitogenome Assembly from Pooled Total DNA Elucidates the Phylogeny of Weevils (Coleoptera: Curculionoidea)
Source: Mol Biol Evol. 2014 May 6;31(8):2223–37. doi: 10.1093/molbev/msu154 (PMC4104315; doi:10.1093/molbev/msu154)
Supplement: Supplementary Data [file supp_msu154_Supplementary_Material_S3-S10.pdf]

SUPPLEMENTARY MATERIAL (S3-S10)

Supplementary Figure S3. Observed nucleotide substitution rates (A) and base frequencies (B) of the six PartitionFinder-selected partitions for the 'all genes dataset'. See Table 2 in the text for partition definitions.

A)

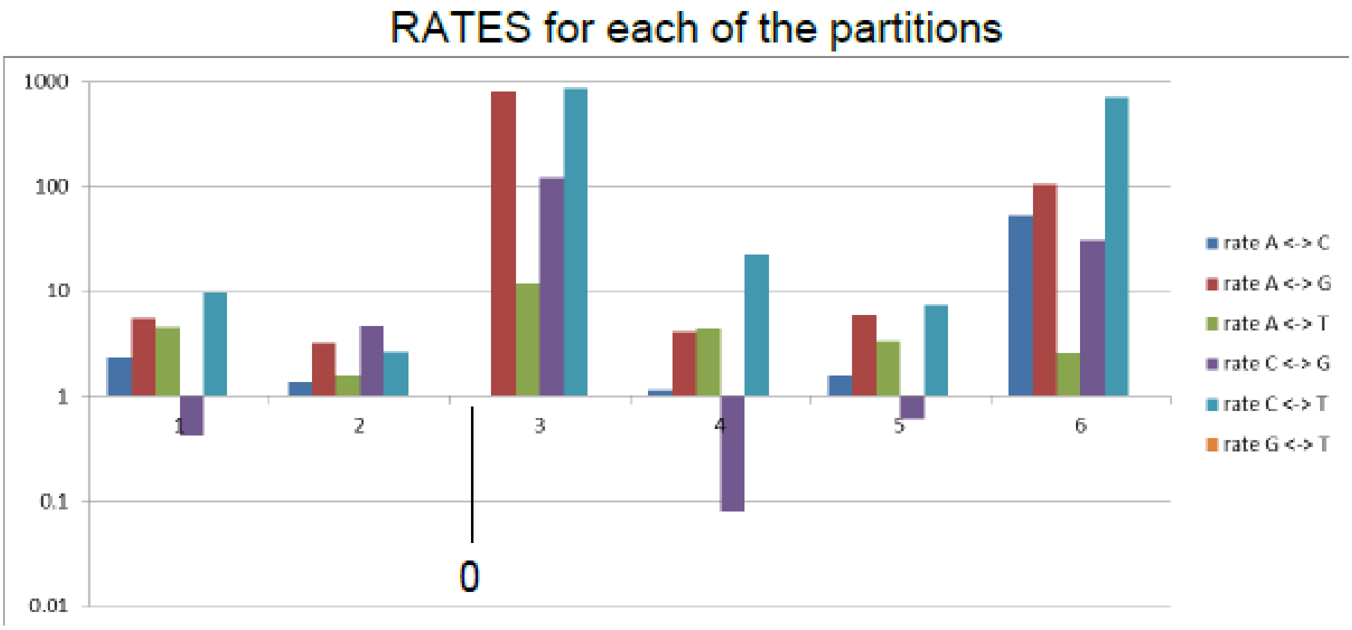

B)

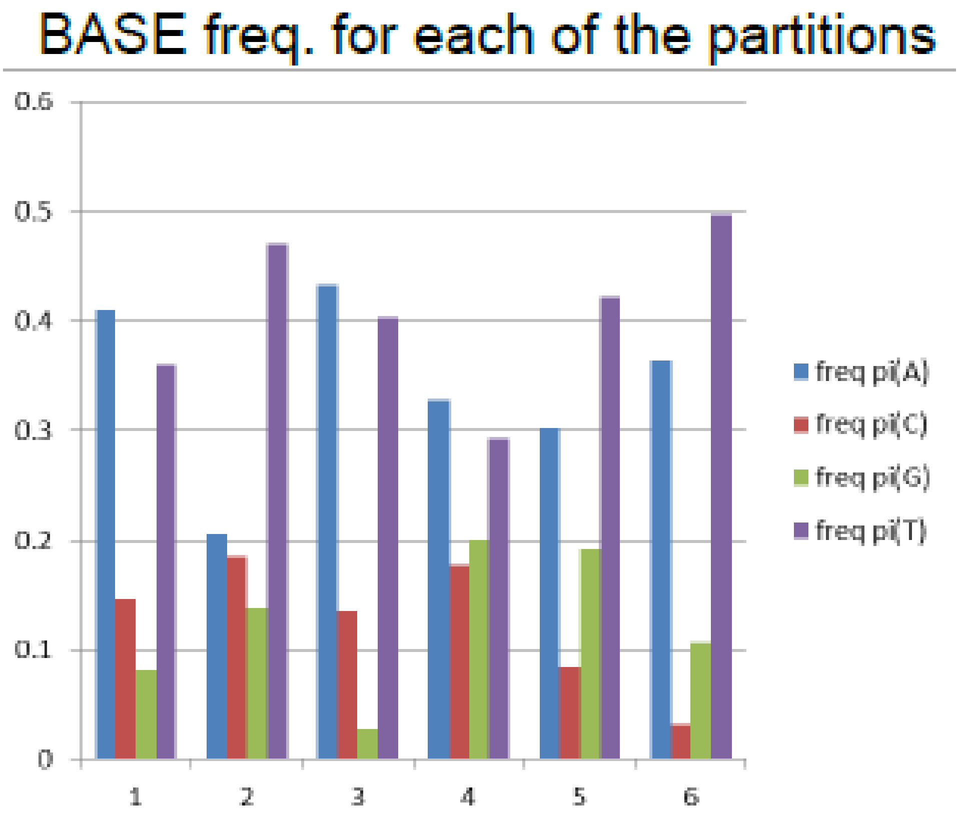

Supplementary Table S4. Final RAxML maximum likelihood optimisation scores for the analyses of each of the 15 datasets. Analyses of datasets containing all genes are shown in grey.

Dataset	Final ML Optimisation Likelihood
All genes partitionjed by gene (A1)	-773731.4614
All genes unpartitioned (A2)	-787772.9784
All genes, PartitionFinder (A3)	-757964.9526
Only protein genes partitioned by gene (B1)	-696122.2766
Only protein genes unpartitioned (B2)	-684161.4211
Only protein genes Partition Finder (B3)	-668479.6459
All genes, protein genes without 3rd codon position, partitioned by gene (C1)	-420952.3613
All genes, protein genes without 3rd codon position, unpartitioned (C2)	-414851.3568
Only proteins genes without 3rd codon position, partitioned by gene (D1)	-328068.0482
Only proteins genes without 3rd codon position, unpartitioned (D2)	-331245.2996
All genes, proteins genes RY coded, partitioned by gene (E1)	-305075.4193
All genes, proteins genes RY coded, unpartitioned (E2)	-310588.2857
Only proteins genes RY coded, partitioned by gene (F1)	-218258.1401
Only proteins genes RY coded, unpartitioned (F2)	-219811.0759
Only protein genes RY coded PartitionFinder (E3)	-218339.2117

Figure S5. Graph of RAxML nodal bootstrap support against branch length of nodes from the root, for the analysis of all 15 concatenated genes under the six partition scheme (dataset A). Nodes with >80% bootstrap are indicated in filled black circles.

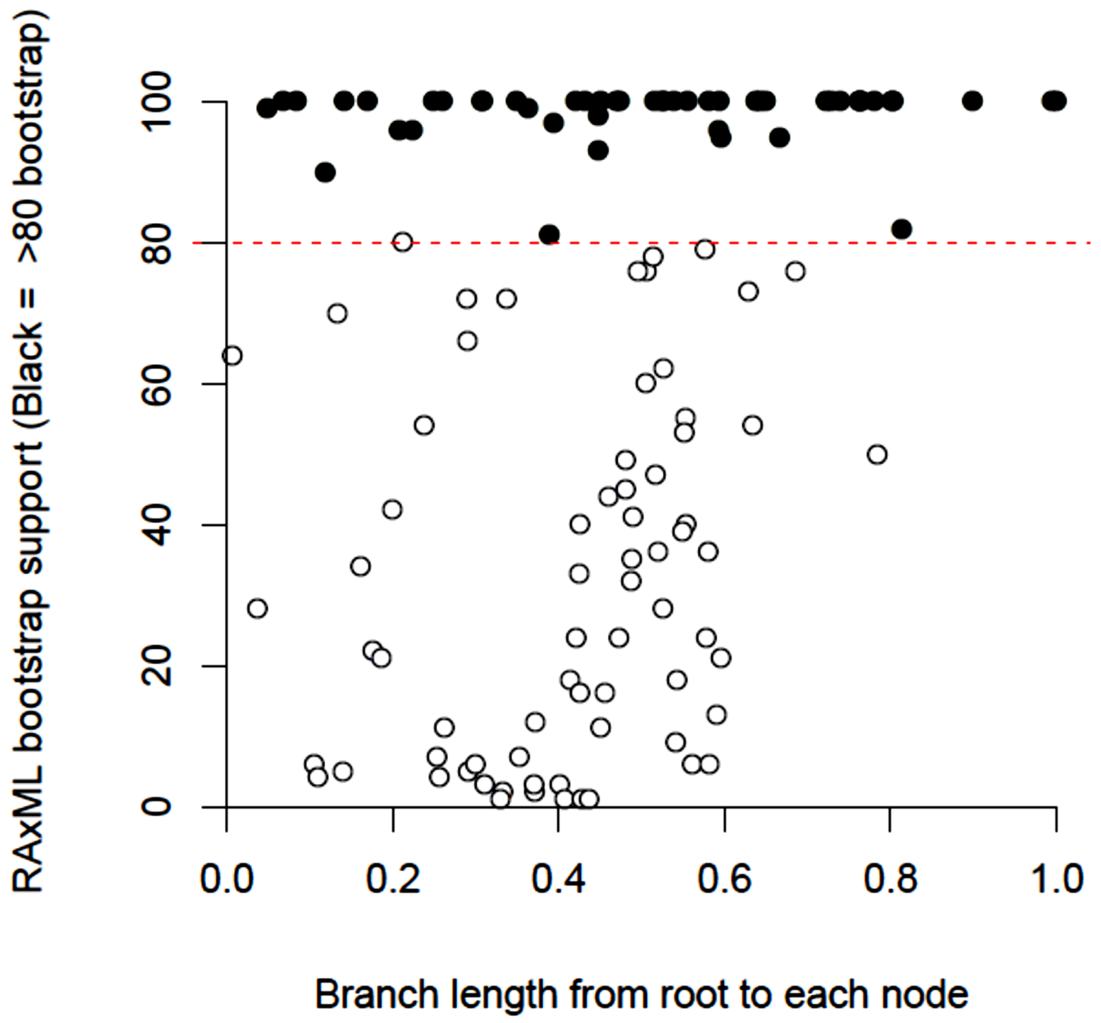

Supplementary Figure S6

Ultrametric RAxML tree of concatenated PCR 'bait' sequences, partitioned by gene.
Numbers indicate nodal support values from 1000 rapid bootstrap replicates.
Family and subfamily prefixes are according to Figure 5 in main text.

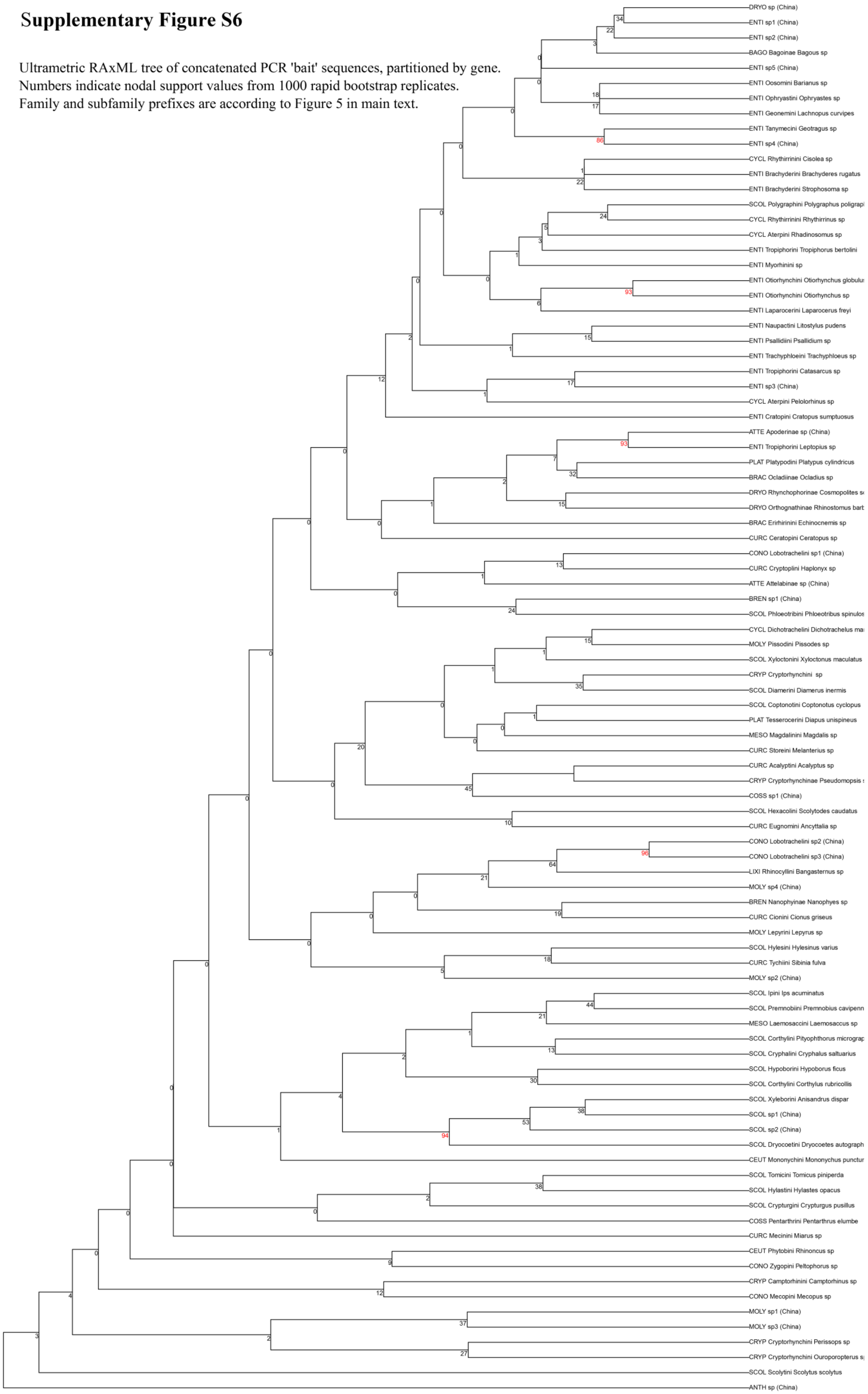

Supplementary Table S7. Test of compositional heterogeneity using the chi-square test measuring significance as implemented in PAUP (Swofford 2001) and using the significance test implemented in Foster (2004) that uses simulations for generating a null model distribution. Tests were performed on all sites and after RY recoding of 1st positions and removing 3rd positions.

Gene	All sites	1st RY coded, 3rd removed	
	PAUP	PAUP	Foster
Concatenated	0	0	0
nad2	0	0.0181846	0
cox1	0	1	0.01
cox2	7.59E-07	1	0.01
atp8	0.188122	0.972425	0
atp6	0	1	0
cox3	0	1	0.4
nad3	5.53E-07	1	0.03
nad5	0	1.06E-13	0
nad4	0	0.893078	0
nad4L	0.00353768	0.998908	0.02
nad6	0	0.0612167	0
cytB	0	1	0.13
nad1	0	1	0.25

Supplementary Figure S8

Ultrametric RAxML tree of the four concatenated reverse-strand transcribed protein-coding genes, partitioned by gene. Numbers indicate nodal support values from 1000 rapid bootstrap replicates. Family and subfamily prefixes are a ccording to Figure 5 in main text.

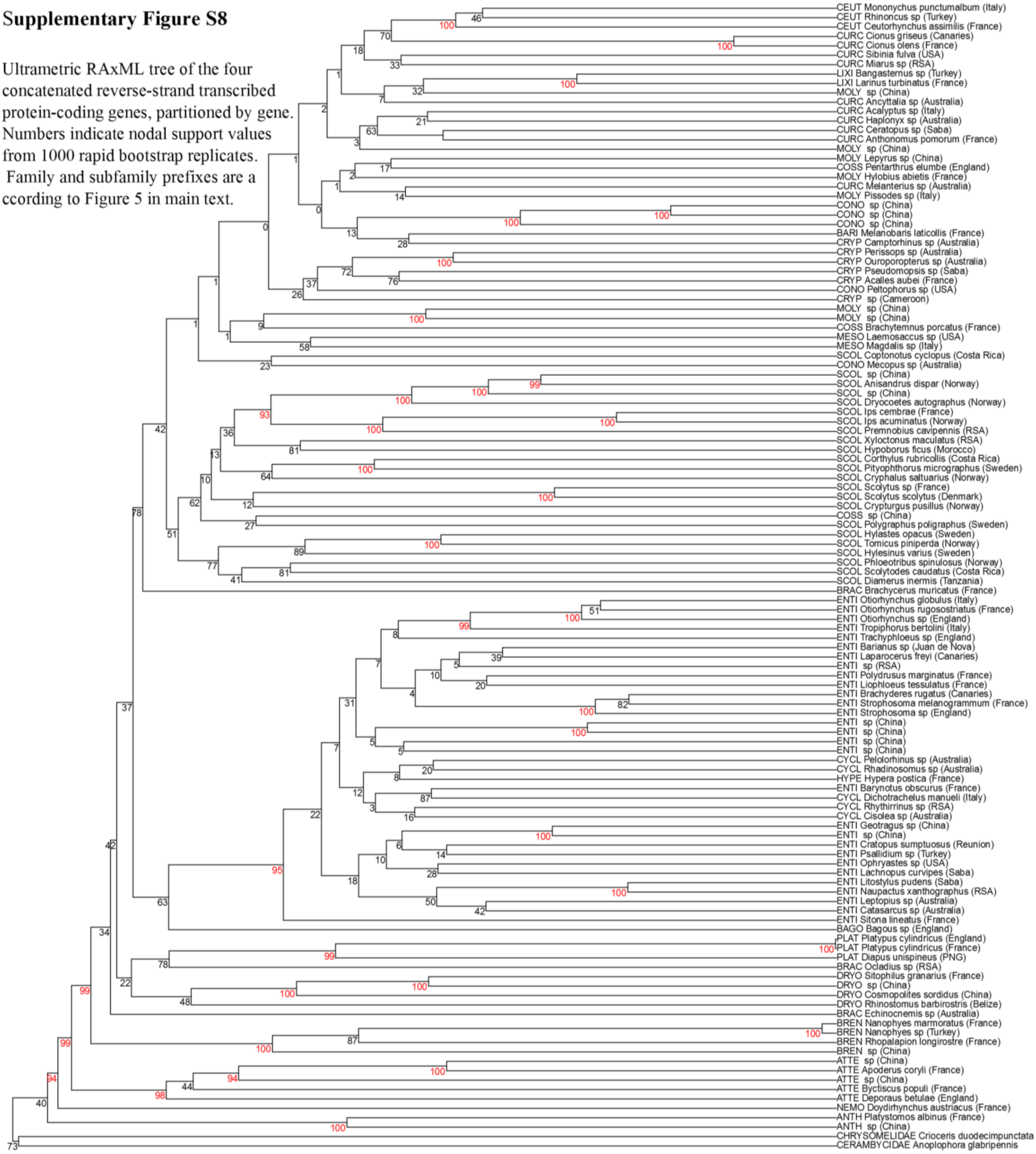

Supplementary Figure S9

Ultrametric RAxML tree of the nine concatenated forward-strand transcribed protein-coding genes, partitioned by gene. Numbers indicate nodal support values from 1000 rapid bootstrap replicates. Family and subfamily prefixes are a ccording to Figure 5 in main text.

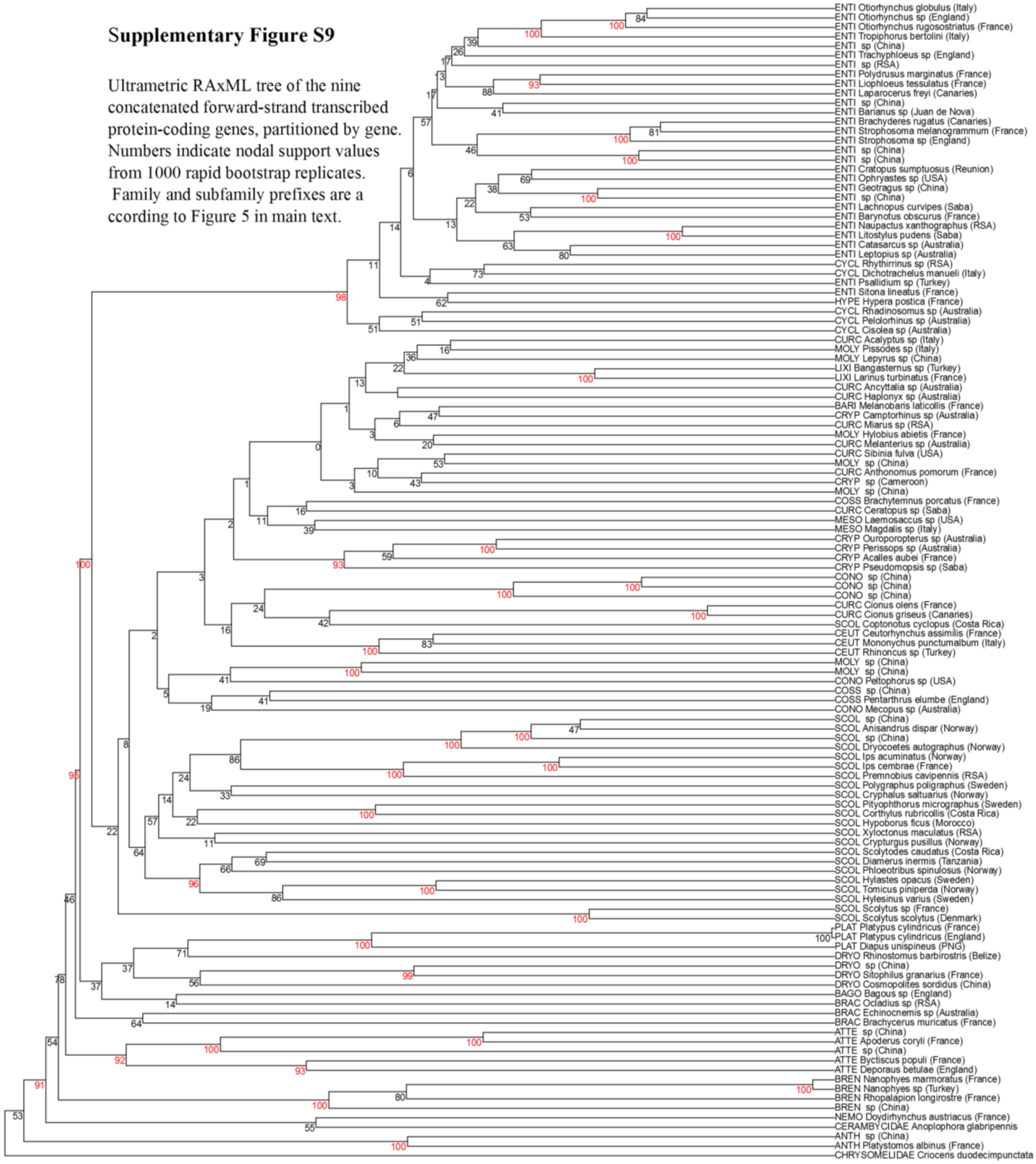

Supplementary Table S10. Primers (A), PCR reaction mixes (B) and cycling conditions (C) for the *cox1*, *cytB* and *rrnL* 'bait' fragment amplifications.

A)

Primer name	Marker	Dir.	Primer sequence 5'=>3'	Anneal Temp.	Length (kbp)
M13REV-FOLbeetF2	*cox1* 5'	Fwd.	CAGGAAACAGCTATGACCTTYTCWACNAAYCAYAARGAYATYGG	52°C	0.7
M13(-21)-FOLbeR2	*cox1* 5'	Rev.	TGTAAAACGACGGCCAGTTANACTTCWGGRTGNCCRAARAAYCA		
M13REV	Sequencing	Fwd.	CAGGAAACAGCTATGACC	50°C	0.7
M13(-21)	Sequencing	Rev.	TGTAAAACGACGGCCAGT		
Jerry	*cox1* 3'	Fwd.	CAACATTTATTTTGATTTTTTGG	53°C	0.8
Stev_pat_R	*cox1* 3'	Rev.	GCACTAWTCTGCCATATTAGA		
SytB_F	*cytb*	Fwd.	TGAGGNCAAATATCHTTYTGAGG	55°C	0.5
SytB_R	*cytb*	Rev.	GCAAATARRAARTATCATTCDGG		
LRJ-12961	*rrnL*	Fwd.	TTTAATCCAACATCGAGG	50°C	0.45
LRJ-12887	*rrnL*	Fwd.	CCGGTCTGAACTCAGATCACGT		
LRN-13398	*rrnL*	Rev.	CGCCTGTTTAACAAAAACAT		

* PCR products amplified with M13REV-FOLbeetF2 were sequenced using M13REV

** PCR products amplified with M13(-21)-FOLbeR2 were sequenced using M13(-21)

B)

PCR Component	***cox1*** **5' X1 (µl)**	***cox13'*** **X1 (µl)**	***cytB*** **X1 (µl)**	***rrnL*** **X1 (µl)**
ddH_2O	15.925	18.15	18.15	18.8
NH_4 buffer X10	2.5	2.5	2.5	2.5
$MgCl_2$ (50mM)	1.5	1.0	1.0	1.0
dNTPs (10mM total/2.5mM each)	2.0	1.0	1.0	1.0
Forward primer (10µM)	1.0	0.625	0.625	0.6
Reverse primer (10µM)	1.0	0.625	0.625	0.6
Taq polymerase	0.075	0.1	0.1	0.1
DNA	1.0	1.0	1.0	1.0
Total volume	25.0	25.0	25.0	25.6

C)

	cox1 5'			*cox1* 3'			*cytB*			*rrnL*		
	**Temp.**	**Time**		**Temp.**	**Time**		**Temp.**	**Time**		**Temp.**	**Time**	
**Initialisation**	95°C	2 mins		94°C	2 mins		94°C	5 mins		94°C	5 mins	
**Denaturation**	95°C	1m	X39	94°C	30s	X35	94°C	30s	X35	94°C	30 s	X35
**Annealing**	52°C	45s		53°C	30s		55°C	30s		50°C	30 s	
**Extension**	72°C	1m		70°C	1m		70°C	1m		72°C	30 s	
**Final extension**	72°C	5 mins		72°C	10 mins		72°C	10 mins		72°C	7 mins	
**Final hold**	10°C	∞		10°C	∞		10°C	∞		10°C	∞	
