# Supplementary material for: Bulk De Novo Mitogenome Assembly from Pooled Total DNA Elucidates the Phylogeny of Weevils (Coleoptera: Curculionoidea)
Source: Mol Biol Evol. 2014 May 6;31(8):2223–37. doi: 10.1093/molbev/msu154 (PMC4104315; doi:10.1093/molbev/msu154)
Supplement: Supplementary Data [file supp_msu154_Supplementary_Table_S2.pdf]

SUPPLEMENTARY MATERIAL

Supplementary Table S2.

Results of identification of mitogenomic assemblies by BLAST searching 'bait' sequences against corresponding cox1 5', cox1 3', cytB and *rrnL* sequences from new assemblies. In grey are indicated successful assembly identifications. Numbers in the bait columns denote the unique identification numbers for the assemblies with the best 'hit' to each bait. Total number of baits available and number of successful 'hits' per sample is shown. For unsuccessful assembly identifications the reason for failure is given.

* Conspecific samples CG343 and CG317 resulted in a single mitogenomic assembly.

SAMPLE	*cox1* 5' BAIT	*cox1* 3' BAIT	*cytB* BAIT	*rrnL* BAIT	TOTAL BAITS	TOTAL BAIT HITS	I.D. SUCCESS?	REASON FOR I.D. FAIL
**CG031**					1	0	**n**	**No bait hits**
**CG052**	181	181	181	181	4	4	**y**	
**CG055**	104	104	104	104	4	4	**y**	
**CG069**	180		180	180	3	3	**y**	
**CG074**	112	112	112	112	4	4	**y**	
**CG205**					3	0	**n**	**No bait hits**
**CG206**	91	91	42	457514	4	4	**n**	**Short assemblies**
**CG210**	156	156	156	156	4	4	**y**	
**CG212**	197		62	74	3	3	**n**	**Short assemblies**
**CG213**	7				3	1	**n**	**Short assemblies**
**CG215**					4	0	**n**	**No bait hits**
**CG220**	183				1	1	**y**	
**CG221**	114	114			2	2	**y**	
**CG222**	150	150		150	3	3	**y**	
**CG223**					1	0	**n**	**No bait hits**
**CG224**					2	0	**n**	**No bait hits**
**CG225**	4	4			2	2	**n**	**Short assemblies**
**CG226**			100-27		1	1	**y**	
**CG227**			89		1	1	**y**	
**CG229**	100-31	100-31	100-31		3	3	**y**	
**CG230**	251327	251327			3	2	**n**	**Short assemblies**
**CG231**					2	0	**n**	**No bait hits**
**CG232**	457690	457690	59	59	4	4	**n**	**Short assemblies**

**CG235**	459182		459182		2	2	**y**	
**CG236**	41				1	1	**n**	**Short assemblies**
**CG237**					3	0	**n**	**No bait hits**
**CG238**	173	173			2	2	**y**	
**CG239**					3	0	**n**	**No bait hits**
**CG240**	177	177			2	2	**y**	
**CG241**					3	0	**n**	**No bait hits**
**CG242**		154	154	154	3	3	**y**	
**CG243**					1	0	**n**	**No bait hits**
**CG244**					4	0	**n**	**No bait hits**
**CG245**					2	0	**n**	**No bait hits**
**CG246**					4	0	**n**	**No bait hits**
**CG247**	43	43	79		3	3	**y**	
**CG248**		100-14			1	1	**y**	
**CG249**					4	0	**n**	**No bait hits**
**CG250**					3	0	**n**	**No bait hits**
**CG252**		51	251254		3	2	**n**	**Short assemblies**
**CG253**	82	82		82	3	3	**y**	
**CG254**		5			1	1	**n**	**Short assembly**
**CG255**					1	0	**n**	**No bait hits**
**CG257**	176	176		176	3	3	**y**	
**CG258**	191	191	200		4	3	**n**	**Short assemblies**
**CG259**					1	0	**n**	**No bait hits**
**CG260**	100-665				2	1	**n**	**Short assemblies**
**CG261**					4	0	**n**	**No bait hits**
**CG263**					1	0	**n**	**No bait hits**
**CG264**					4	0	**n**	**No bait hits**
**CG265**					1	0	**n**	**No bait hits**
**CG266**					3	0	**n**	**No bait hits**
**CG267**			84		2	1	**n**	**Short assemblies**
**CG268**	100-2	100-2	100-2	100-2	4	4	**y**	
**CG269**	214	214		73	4	3	**n**	**Short assemblies**
**CG270**	100-337	100-337	459069		4	3	**n**	**Short assemblies**

**CG271**		44	44	44	3	3	**y**	
**CG272**	96	96	96	96	4	4	**y**	
**CG274**					4	0	**n**	**No bait hits**
**CG275**					3	0	**n**	**No bait hits**
**CG276**					4	0	**n**	**No bait hits**
**CG277**	201	201			4	2	**n**	**Short assemblies**
**CG278**	203	203	189		3	3	**n**	**Short assemblies**
**CG279**		41	60	457037	4	3	**n**	**Short assemblies**
**CG280**		31	94		3	2	**n**	**Short assemblies**
**CG281**		67	14		4	2	**n**	**Short assemblies**
**CG282**	130	130	130	130	4	4	**y**	
**CG283**		127		127	2	2	**y**	
**CG284**	100-26	100-26	100-26		3	3	**y**	
**CG285**	182	182			2	2	**y**	
**CG286**			13		3	1	**n**	**Short assemblies**
**CG287**					3	0	**n**	**No bait hits**
**CG288**	100-6		100-6		2	2	**y**	
**CG289**	120	120	458630	458630	4	4	**y**	
**CG290**					1	0	**n**	**No bait hits**
**CG291**					1	0	**n**	**No bait hits**
**CG293**	146		146		2	2	**y**	
**CG295**	145	145	145		3	3	**y**	
**CG296**	175	175			2	2	**y**	
**CG297**	153	153	153		3	3	**y**	
**CG298**	147	147	147		3	3	**y**	
**CG299**	25	25			4	2	**n**	**Short assemblies**
**CG300**	134B	134B	134B	134B	4	4	**y**	
**CG301**	113	113	113	113	4	4	**y**	
**CG302**					3	0	**n**	**No bait hits**
**CG303**					3	0	**n**	**No bait hits**
**CG304**			195	195	4	2	**n**	**Short assemblies**
**CG305**		148			1	1	**y**	
**CG306**		458889	458889	129	3	3	**y**	

**CG307**		142	142	142	3	3	**y**	
**CG308**					1	0	**n**	**No bait hits**
**CG309**	108	108	108	100-231	4	4	**y**	
**CG310**		34	40		4	2	**n**	**Short assemblies**
**CG311**	95	95	95		4	3	**y**	
**CG312**	144	144	144	144	4	4	**y**	
**CG313**				147	1	1	**n**	**Ambiguous 16S hits**
**CG314**				147	4	1	**n**	**Ambiguous 16S hits**
**CG315**	126	126	126	126	4	4	**y**	
**CG316**		50			3	1	**n**	**Short assemblies**
**CG317**		81	81	81	3	3	**y**	
**CG318**					3	0	**n**	**No bait hits**
**CG319**				165	1	1	**y**	
**CG320**					2	0	**n**	**No bait hits**
**CG321**			100-94		1	1	**y**	
**CG322**			123		1	1	**y**	
**CG323**			80	80	2	2	**y**	
**CG324**				110	1	1	**y**	
**CG325**	117	117	117		3	3	**y**	
**CG326**					2	0	**n**	**No bait hits**
**CG327**		206			4	1	**n**	**Short assemblies**
**CG328**			163	163	2	2	**y**	
**CG329**			246528		1	1	**n**	**Short assemblies**
**CG330**			100-25	100-25	2	2	**y**	
**CG331**	119	119	119	119	4	4	**y**	
**CG332**		100-28	100-28	100-28	3	3	**y**	
**CG333**			36		1	1	**n**	**Short assembly**
**CG334**					1	0	**n**	**No bait hits**
**CG335**			75		1	1	**y**	
**CG336**		160			1	1	**y**	
**CG337**					1	0	**n**	**No bait hits**
**CG338**			10		1	1	**n**	**Short assembly**
**CG339**		149	149	149	3	3	**y**	

**CG340**		141	141	141	3	3	**y**	
**CG341**		152	152		2	2	**y**	
**CG342**		162	162	162	3	3	**y**	
**CG343**		81	81		2	2	**y***	
**CG344**			101	101	2	2	**y**	
**CG346**	161		161	161	3	3	**y**	
**CG347**		178	178		2	2	**y**	
**CG348**					3	0	**n**	**No bait hits**
**CG349**			168	168	2	2	**y**	
**CG350**		164	164	164	3	3	**y**	
**CG351**			56		2	1	**y**	
**CG352**			115	115	2	2	**y**	
**CG353**			457326		1	1	**n**	**Short assemblies**
**CG354**		139	139	139	3	3	**y**	
**CG355**		107	143	143	3	3	**y**	
**CG412**					1	0	**n**	**No bait hits**
**CG414**					3	0	**n**	**No bait hits**
**CG415**		102	185		2	2	**y**	
**CG418**					1	0	**n**	**No bait hits**
**CG419**			124	124	2	2	**y**	
**CG420**		172		172	2	2	**y**	
**CG421**					3	0	**n**	**No bait hits**
**CG422**		76	190	190	3	3	**y**	
**CG423**		137	137	137	3	3	**y**	
**CG424**		116	116		2	2	**y**	
**CG425**		151	151		2	2	**y**	
**CG426**		158	158		2	2	**y**	
**CG427**		210			3	1	**n**	**Short assemblies**
**CG428**			169	169	2	2	**y**	
**CG429**		118			1	1	**y**	
**CG430**		93			3	1	**n**	**Short assemblies**
**CG431**		109	109	109	3	3	**y**	
**CG432**			193	193	3	2	**n**	**Short assemblies**

**CG434**			157		1	1	**y**	
**CG435**		155	155		2	2	**y**	
**CG436**		77	77	77	3	3	**y**	
**CG437**		122	122	122	3	3	**y**	
**CG438**		136	136		2	2	**y**	
**CG439**			184	184	2	2	**y**	
**CG440**					1	0	**n**	**No bait hits**
**CG441**				159	1	1	**y**	
**CG442**		174	174		2	2	**y**	
**CG443**		457110	100-215	250606	3	3	**n**	**Short assemblies**
**CG444**				135	1	1	**y**	
**CG445**		170		170	2	2	**y**	
**CG446**					1	0	**n**	**No bait hits**
**CG447**	61			52	2	2	**n**	**Non-weevil**
**CG448**					2	0	**n**	**No bait hits**
**LAP007**	98	98	98		3	3	**y**	
**N28**	134A			134A	2	2	**y**	
**T-Reu3834**		140	140	140	3	3	**y**	
